# Hydro-alcoholic extract of *Matricaria recutita* exhibited dual anti-spasmodic effect via modulation of Ca^2+^ channels, NO and PKA_2_-kinase pathway in rabbit jejunum

**Published:** 2017

**Authors:** Hamidreza yazdi, Akhtar Seifi, Shima Changizi, Vahid Khori, Fatemeh Hossini, Ali Davarian, Yahya Jand, Ayesheh Enayati, Masumeh Mazandarani, Fateme Nanvabashi

**Affiliations:** 1 *Ischemic Disorders Research Center, Golestan University of Medical Sciences, Gorgan, Iran*; 2 *Department of Botany, Islamic Azad University, Gorgan, Iran*

**Keywords:** Matricaria, Cholinergic Agents, Histamine Agents, Jejunum, Rabbit, Nitric Oxide

## Abstract

**Objective:**

Several studies have shown the antispasmodic activity of *Matricaria* *recutita* without detailing the underlying mechanism(s). The present study was designed to determine whether the antispasmodic mechanisms of *M. recutita* extract mediated via histaminergic/cholinergic receptors, Ca^2+^channels, activation of PKA_2 _and NO release in isolated rabbit jejunum.

**Materials and Methods:**

The concentration- dependent (3 × 10^-3^–1.3 × 10^-2^ mg/ml) antispasmodic effect of the hydro-alcoholic extract of *M. recutita *flowers was studied in isolated rabbit jejunum. The isolated jejunum preparations were divided into seven groups, including the pharmacological probes that modulate cholinergic, histaminergic, and nitrergic receptors, as well as PKA_2_.

**Results:**

*M. recutita* inhibited spontaneous smooth muscle contractility of the jejunum in a concentration-dependent manner (3 × 10^-3^–1.3 × 10^-2 ^mg/ml) and reduced both K^+^- and Ca^2+^-induced contractions, which is similar to the effect of verapamil. The antispasmodic effect of *M. recutita *was inhibited by H89 (a PKA_2 _inhibitor). The myorelaxant effect of *M. recutita *increased in the presence of ACh/His and H89.

**Conclusion:**

*M. recutita *evoked antispasmodic and spasmolytic effects mediated through different signaling pathways. Our results have shown this dual inhibitory effect is mediated by blocking Ca^2+^ channels, activating His and ACh receptors, releasing NO, and activating PKA_2_.

## Introduction

*Matricaria recutita* (German chamomile, Asteraceae *Matricaria chamomilla* L. var. recutita) is one of the most abundant annual medicinal herbs in the world. It has been used as an anti-inflammatory drug, an analgesic for gastrointestinal pain, as well as an antiseptic, an anti-microbial, and a sedative remedy in traditional medicine in Europe and Asia (McKay, et al., 2006 ▶). Clinical and animal studies have shown that *M. recutita* extract is effective as a smooth muscle relaxant (Ammon, et al., 2006 ▶; mazandarani, et al., 2011 ▶). It contains potentially active chemical constituents such as terpenoids, flavonoids, coumarins, and spiroethers. However, chamomile’s main active constituents are chamazulene, apigenin and bisabolol. It seems that flavonoids are the most potent antispasmodic products of *M. recutita*, while its other important components such as bisabolol, chamazulene, and α–bisabolol have anti-inflammatory effects (Abad, et al., 2011 ▶).

 Flavonoids are not produced by the human body and, generally, daily diet is the main available source for them. Different disciplines such as chemistry, biochemistry and pharmacy have provided evidence of the vital role of flavonoids (Peng, et al., 2003 ▶).

Forster and Achterrath-Tuckermann studied the antispasmodic effects of *M. recutita* and its flavonoids in guinea-pig ileum. They found that the antispasmodic effect of *M. recutita* is 91% greater than that of papaverine, a myorelaxant alkaloid from the poppy plant (Achterrath-Tuckermann, et al., 1980 ▶; Forster, et al., 1980 ▶). Apigenin is a major flavonoid found in *M. recutita* and it is recognized for its antiphlogistic, antispasmodic and antibacterial effects (Guenwaldr, et al., 1998 ▶; Murti, et al., 2012 ▶). In terms of gastrointestinal effects, flavonoids decrease smooth muscle contractions, possibly by interfering with calcium movement through cell membranes (Di Carlo, et al., 1999 ▶).

Among numerous studies on the antispasmodic mechanism of *M. recutita*, few have considered downstream signaling cascade of this plant, especially NO and PKA_2_ pathways. In the present study, we hypothesized that *M. recutita *induces its anti-spasmodic effect via several signaling cascades including Ca^2+^ channel blockade, NO release, ACh/His receptors, and PKA_2_ activation. Here, we investigated 1) the concentration-dependent anti-spasmodic effect of *M. recutita *in the presence of contractions induced by K^+^ and Ca^2+^, 2) anti-spasmodic mechanism of *M. recutita *in the presence of the ACh/His blocker, and NOS and PKA_2 _inhibitors.

## Materials and Methods

**Drugs**

Acetylcholine chloride (ACh), atropine sulfate, histamine dihydrochloride (His), cetirizine, L-NAME, l-arginine, potassium chloride, calcium chloride, verapamil hydrochloride, and H-89 were obtained from Sigma Chemicals Company, St. Louis, USA. All drugs were dissolved in distilled water and diluted in fresh Tyrode’s solution.

The hydro-alcoholic extract of *M. recutita* was obtained from Niak Pharmaceutical Company, Gorgan, Iran.

**Plant material and phytochemical analysis of extract**

All phytochemical procedures were performed as previously described (mazandarani et al., 2011 ▶). Fresh *M. recutita* flowers were harvested from a farm by Niak Pharmaceutical Company (Golestan province, Iran) in May 2014. The plant was identified in the herbarium of Azad University, Gorgan, Iran (RCMP-0100 code). *M. recutita* flowers were immediately separated, dried in an oven at 40 ºC, milled to a coarse powder (Mesh. 100) and extracted by the maceration method over 6 days using ethanol 70%. This hydro-alcoholic extract was then filtered and dried in vacuum evaporator (at 40–50 ºC). The percentage dried weight of the extract was 30.80% (Mohammedi, et al., 2011 ▶).

To analyze the phenolic and flavonoid contents of the extract, a 2.5 g sample of the dried extract was used. For each treatment, analyses were performed in triplicate.

**Determination of total phenolic and flavonoid content**

The total phenolic content of the extract was determined by the Folin–Ciocalteu method. Briefly, 0.2 ml of the extract was mixed with 0.5 ml of Folin–Ciocalteu reagent, 1.5 ml of 20% Na_2_CO_3_ (Merck, Germany) solution, and 7.8 ml of distilled water. The sample was left for 2 hr at room temperature and its absorbance at 765 nm was determined using a UV-visible spectrophotometer (Shimadzu, Japan). The result was expressed in terms of gallic acid (Merck, Germany) equivalent mg/g dry weight of the extract. The amount of flavonoids was determined with the colorimetric method by using aluminum chloride hexahydrate (AlCl_3_.6H_2_O; Merck, Germany). Absorbance at 395 nm was read using a UV-vis spectrophotometer (Shimadzu 1601). The result was expressed in terms of apigenin (Merck, Germany) equivalent mg/g dry weight of the extract. The total phenolic and flavonoid contents of the extract were 17.2 ± 1.3% and 5.6 ± 0.4%, respectively.

**Animals**

In this study, we used male New Zealand white rabbits weighing 1.8–2.5 kg (Pasteur Institute of Iran, Iran). These rabbits were housed in the animal house of Golestan University of Medical Sciences, Iran, under standard laboratory conditions and provided with animal food pellets and free access to water. However, food supply was ceased 12 hr prior to experimentation. The study was approved by the Institutional Animal Ethics Committee and carried out according to Golestan University of Medical Sciences’ guidelines for the use and care of laboratory animals.

**Isolated tissue preparations**

The anti-spasmodic activities of *M. recutita* were studied using isolated tissue from rabbit jejunum. The experiments were performed using jejunum sections taken from five rabbits. The rabbits were anaesthetized with sodium pentobarbital (40 mg/kg), and heparin (200 IU/kg) was used as an anticoagulant. The abdomen was exposed and opened. Several pieces of 2cm-long of jejunum were taken, cleaned, and transferred into a Petri dish containing Tyrode’s solution, where it was detached from the mesenteric attachments. Each jejunum segment was mounted vertically (with open lumen) under a resting tension of 0–20 g in 25 mL tissue baths containing Tyrode’s solution, connected to an isometric force transducer (N.TRI 202P, AD Instruments Pty Ltd., Sydney, Australia), and allowed to equilibrate for 20 min. The tissue-bathing medium was maintained at 37 °C (pH 7.4), and gassed with 95% oxygen and 5% carbon dioxide. The composition (mM) of the Tyrode’s solution used herein was as follows: NaCl (136.9 mM), KCl (2.7 mM), CaCl_2_ (1.8 mM), MgCl_2_ (1 mM), NaHCO_3_ (11.9 mM), NaH2PO_4_ (0.42 mM), and glucose (5.5 mM). For determination of Ca^2+^ channel blocking (CCB) activity, K^+^ (50 mM) was used to induce stable contraction of the preparations, as previously described (Farre, et al., 1991 ▶). When stable and sustained contraction was achieved, verapamil was added cumulatively to obtain the concentration-dependent inhibitory response (Van Rossum, 1963 ▶). To confirm the Ca^2+^ antagonist activity of the extract, the tissue was allowed to stabilize in normal Tyrode’s solution, which was then washed out with Ca^2+^-free Tyrode’s solution containing EDTA (0.1 mM) for 20 min. This solution was then replaced with K^+^-rich and Ca^2+^-free Tyrode’s solution of the following composition: KCl 50 mM, NaCl 91.04 mM, MgCl_2_ 1.05 mM, NaHCO_3_ 11.90 mM, NaH_2_PO_4_ 0.42 mM, glucose 5.55 mM, and EDTA 0.1 mM. Following incubation for 20 min, concentration-response curves (CRCs) control of Ca^2+^ was obtained. When the CRCs of Ca^2+^ were found to be superimposable (usually after two cycles), the tissue was pretreated with the extract for 30 min to test the CCB effect. The CRCs of Ca^2+^ were reconstructed at different concentrations of *M. recutita* and other drugs.

**Statistical analysis**

All results are presented as mean ± SEM. All data were tested for normality before applying the statistical tests. Two groups of experimental data were compared using Student’s t-test. Concentration-dependent changes were examined using repeated-measures analysis of variance (ANOVA) and Scheffe contrasts. Changes in force were presented as a percentage of the maximal response induced by agonists (Acetylcholine, Histamine, KCL, and Ca^2+^). The EC_50_ values were obtained from cumulative concentration-effect curves via non-linear regression analysis of data points from individual concentration-effect curves plotted using GraphPad Prism 5 (GraphPad Software, Inc., CA, USA). A probability of 5% or lower was considered statistically significant.

## Results

**Figure 1:**
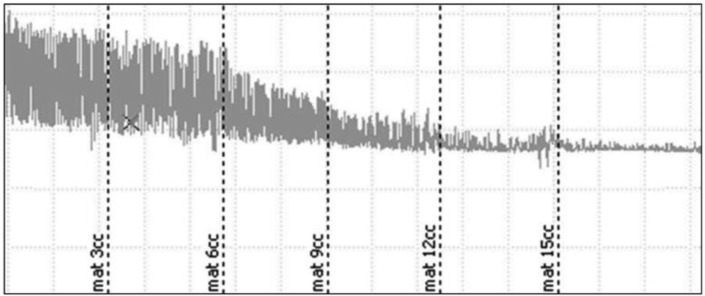
Concentration-dependent effects of *M. recutita* on the spontaneous contractions in isolated rabbit jejunum preparation (are presented as mean ±SEM, n=5). Mat: *Matricaria recutita*

**Figure 2:**
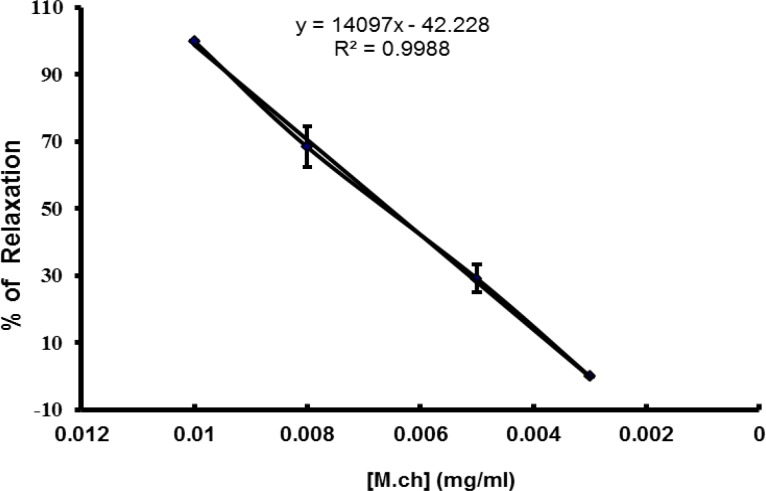
Inhibitory effect of M. recutita on spontaneous and high K+-induced contractions in isolated rabbit jejunum preparations. (Data are presented as mean±SEM, n=5

Phytochemical analysis of the flavonoid extract of *M. recutita* indicated the presence of quercetin, apigenin, luteolin, and patuletin. In the concentration-dependent model, the hydro-alcoholic extracts of *M. recutita* (3 × 10^-3^–13 × 10^-3^ mg/ml) inhibited spontaneous jejunum contractions, with an EC_50_ of 6.3 × 10^–3^ mg/ml (n = 5) (Figure 1). K^+^ (50 mM)-induced contractions of jejunum smooth muscle were inhibited by the *M. recutita *extract (EC_50_ = 6.5 × 10^-3^ mg/ml; n = 5) (Figure 2). *M. recutita* had nearly the same EC_50_ in the presence or absence of smooth muscle contraction (EC_50_ = 6.3 × 10^-3^ mg/ml).

**Figure 3:**
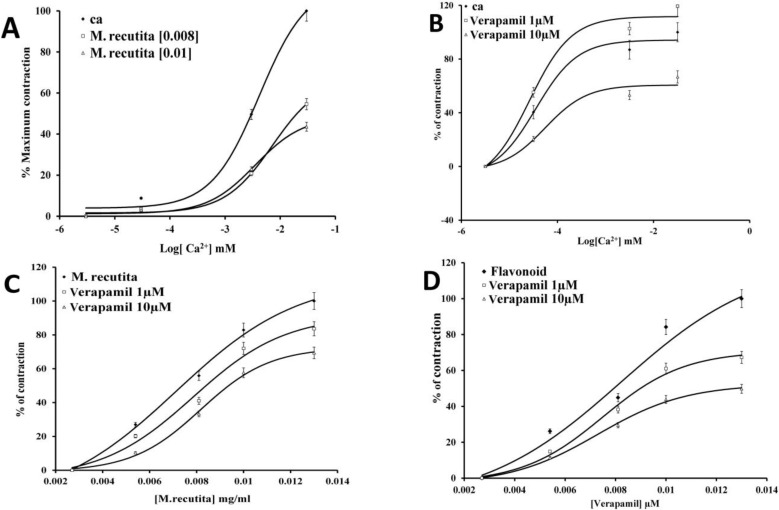
(a) and (b) show the concentration–response control curves of Ca2+ in the absence and presence of increasing concentrations of M. recutita and verapamil, respectively, constructed in Ca2+-free medium. (c) and (d) show the anti-spasmodic activity of M. recutita and flavonoids, respectively, in the presence of verapamil (Data are presented as mean ±SEM, n=5

To determine whether the mechanism of antispasmodic activity of *M. recutita* was mediated through calcium channel blockade, jejunum was stabilized in a Ca^2+^-free Tyrode’s solution to inhibit smooth muscle contractions. Then, Ca^2+^ was added cumulatively (3 × 10^-3^–30 mM) in the presence and absence of *M. recutita *(8 × 10^-3^–1 × 10^-2 ^mg/ml), wherein the extract caused a rightward shift in the Ca^2+ ^concentration–response curves with EC_50_ = 3 × 10^-3^ and 7 × 10^-3^ (mM), respectively (Figure 3a). The inhibitory actions of the total extract and the flavonoid fraction were similar to that of verapamil (1 - 10 µM) with EC_50_ = 2.62 × 10^-5^ and 5.54 × 10^-5^ µM, respectively (n = 5), (Figures 3b, c and d).

**Figure 4:**
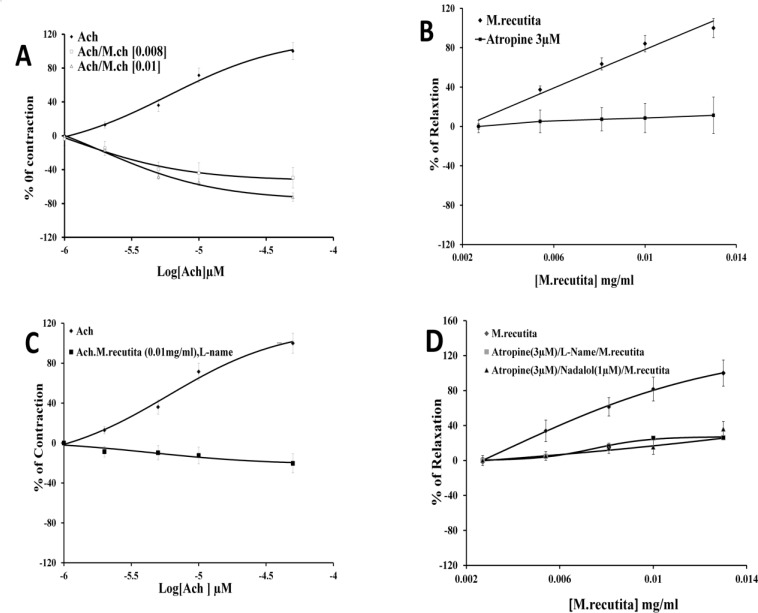
(a) Comparison of dose–response curves of *M. recutita* in the presence of acetylcholine-induced contraction of isolated rabbit jejunum, (b) shows depressing effect of L-NAME on *M.recutita* in the presence of acetylcholine, (c) and (d) show effect of *M.recutita* extract in the presence of atropine and nadolol respectively ( Data are presented as mean ±SEM, n = 5

Acetylcholine (ACh) caused a rapid increase in contractions at concentrations of 1–50 µM (EC_50_ = 5.9 × 10^-6^ µM). Pretreatment of the intestinal tissue with *M. recutita* extract (0.01 mg/ml) reversed the tissue’s response to ACh with EC_50_ = 1.8 × 10^-6^ mg/ml, where ACh inhibited basal jejunum contractions in a concentration-dependent manner (Figure 4a). Atropine (3µM) prevented the inhibitory effects of *M. recutita *in the presence of ACh (Figure 4b). The inhibitory effect of *M. recutita *in the presence of ACh was not observed in the presence of L-NAME, nadolol (EC_50_ = 4.42 × 10^-6^ mg/ml) (n = 5) (Figures 4c and d).

**Figure 5:**
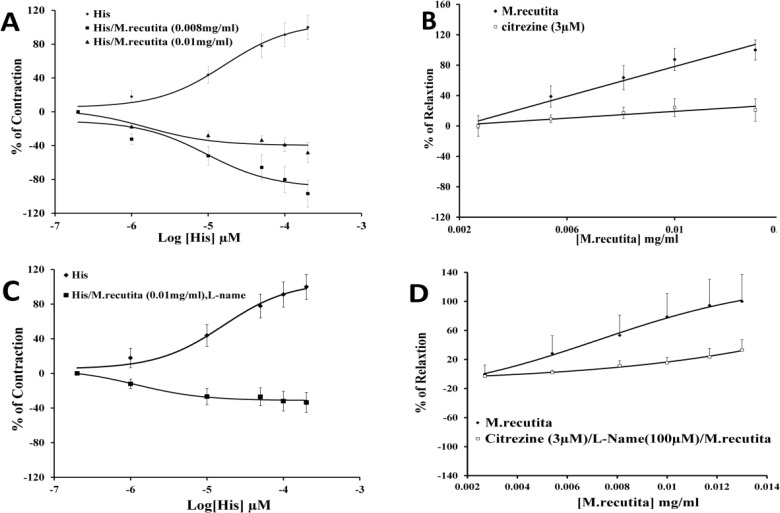
a) and (b) show the effect of *M. recutita* on histamine-induced contraction of isolated rabbit jejunum in the absence and presence of L-NAME, respectively. (c) and (d) show the effect of *M.recutita* extract with cetirizine in the absence and presence of L-NAME, respectively (Data are presented as mean ±SEM, n = 5

Histamine (0.2–200 µM) increased the basal contractions of smooth muscle in a concentration-dependent manner. *M. recutita* (0.01 mg/ml) caused a downward shift of the dose-response curve of histamine (Figure 5a). Similarly, the inhibitory effects of *M. recutita *on smooth muscle contraction were prevented by cetirizine (3 µM) (Figure 5b). Pretreatment of the extract with L-NAME abolished the inhibitory response of *M. recutita* (0.01 mg/ml) in the presence of histamine (EC_50_ = 1.19 × 10^-6^ mg/ml) (Figures 5c and d) (n = 5).

L-arginine (100 mM) significantly reduced the antispasmodic effect of *M. recutita*. The inhibitory effect of *M. recutita* (3 × 10^-3^–13 × 10^-3^ mg/ml) on the intestinal smooth muscle was abrogated in the presence of L-name and L-arginine (EC_50_ = 1.99 × 10^-8^ mg/ml and 388.9 mg/ml), respectively (n = 5). (Figures 4c and d and Figures 5c and d)

In particular, to determine the possible involvement of PKA_2_ and NO in the anti-contractility effects of *M. recutita*, we used H89, a specific inhibitor of PKA_2_. Applying H89 (1 µM) in the presence of *M. recutita* increased tissue relaxation by about 53%. Furthermore, addition of histamine increased the inhibitory activity of the extract by about 38%, whereas ACh and L-arginine reduced its antispasmodic activity by 16% and 10%, respectively (Figures 4c and d and Figures 5c and d).

## Discussion

In the present study, we demonstrated the anti-spasmodic and spasmolytic (relaxant) effects of the flavonoid components of *M. recutita* (3 × 10^-3^–1.3×10^-2^ mg/ml) in isolated rabbit jejunum. *M. recutita* evoked a spasmolytic effect on K^+^-induced spontaneous contractions, suggesting that calcium channels are involved in its spasmolytic actions. This effect was abrogated by L-NAME and amplified by H89. Consistent with this, the spasmolytic effect of* M. recutita* was mediated through the NO/PKA_2_ pathway. Our study showed that the spasmolytic effect of *M. recutita* was mediated through NO-activated cholinergic and histaminergic receptors. 

In the present study, *M*. *recutita* prevented contractions induced by high potassium concentration, suggesting the possible role of voltage-dependent Ca^2+^ channels in its mechanism of action. It is well-documented that KCl induces smooth muscle contractions by activating calcium channels (Cortes, et al., 2006 ▶). Therefore, this data indicated the indirect Ca^2+^ channel blocking activity of *M. recutita* and its flavonoid fraction. Consistent with this, several studies have shown the potential Ca^2+^ channel blocking activity of *M. recutita *extract (Achterrath-Tuckermann et al., 1980 ▶; Forster et al., 1980 ▶; Rotondo, et al., 2009 ▶). In contrast, verapamil did not prevent the antispasmodic activity of *M. recutita*, suggesting that the *M. recutita* extract has a wide range of biological activity.* M. recutita *at low doses could augment the effects of verapamil and this is irrespective to indirect reaction with Ca^2+^channels. 

Moreover, atropine and cetirizine blocked the spasmolytic effect of *M. recutita*, which probably shows a direct receptor-mediated effect of* M. recutita.* This possibly indicates direct mediatory roles of ACh/His receptors in the mechanism of action of *M. recutita*. In addition, several studies have proved the direct role of ACh and His in the muscle relaxant activity of *M. recutita *(Hagelauer, et al., 2006 ▶; Heinle, et al., 2006 ▶). Taken together, *M. recutita*-induced muscle relaxation, was partly mediated through ACh and His receptors. 

Our data have shown that an NOS inhibitor (L-NAME) abrogated the relaxing effect of *M. recutita*. Along the lines of our results, Achterrath-Tuckermann *et al.* reported that *M. recutita* induced a greater anti-contractility effect in the presence of NO donors (Achterrath-Tuckermann et al., 1980 ▶). We hypothesized that the muscle relaxant effect of *M. recutita* is mediated by the release NO and activation of ACh/His receptors. Indeed, a study has demonstrated that ACh/His-induced smooth muscle contraction is mediated by a NO-dependent pathway (Grifoni, et al., 2000 ▶). Taken together, in the present study, *M. recutita-*induced smooth muscle relaxant activity was mediated via NO release and ACh/His receptors. Precise muscle relaxant signaling mechanism of *M. recutita* is yet to be determined. 

Furthermore, our findings showed that the inhibitory effect of *M. recutita *on jejunum contraction is abrogated by L-arginine, due to an unknown mechanism. Applying of high concentrations of L-arginine can induce acidosis and this indirectly prevents the *M. recutita *action. In a separate group of the experiment, we adjusted the pH of medium and interestingly L-arginine showed the previous profile of action. Consistent with this data, few studies have shown that the inhibitory effect of *M. recutita *on the contractile tone of smooth muscle was restored by L-arginine, indicating a controversial dual role of NO as an inhibitor or a stimulator (Michel, et al., 2011 ▶; Westfall, et al., 2011 ▶). Consistent with this, it can be concluded that the controversial dual role of NO in the signaling cascades of *M. recutita *on intestinal motility depends on the basal contractility state of smooth muscle, and it is observable as a spasmolytic effect on contracting smooth muscle and a tonicising effect on relaxed muscle. Ammon et al indicated the dual activity of STW5 in both hypotonic and spastic states of isolated guinea pig ileum(Ammon et al., 2006 ▶). Taken together, NO and ACh/His receptors involved in the signaling cascades of *M. recutita* intestinal smooth muscle contractility 

A previous study demonstrated that NO-evoked smooth muscle relaxation was induced by increasing cGMP and PKA_2 _(Hobbs, et al., 1996 ▶). To determine the possible role of PKA_2_ modulation in NO-induced smooth muscle relaxation, we analyzed the dual anti-spasmodic effects of *M. recutita* in the presence of a selective PKA_2_ inhibitor.

Our results showed that the effects of *M. recutita* were mediated directly or indirectly via PKA_2_. In fact, the inhibitory effects of *M. recutita* on jejunum contractions were augmented by H-89, indicating that the inhibitory effect of *M. recutita* was mediated through PKA_2_ activation. In agreement with our data, a study has shown that the spasmolytic effect of *M. recutita* is mediated by the inhibition of cAMP phosphodiesterase (Maschi, et al., 2008 ▶). In addition, the muscle relaxant function of GI smooth muscle tone was regulated by cAMP-PDE pathway and the flavonoid fraction of *M. recutita* inhibits cAMP activity (Murthy, 2006 ▶). Therefore, our data indicated that the PKA_2_-cAMP transduction pathway was involved in the inhibitory effect of *M. recutita* on smooth muscle contraction. 

Consistent with our results, it has been proven that the gastrointestinal (GI) smooth muscles are synchronized by different mechanisms including the histaminergic, cholinergic, nitrergic pathways to stimulate PKA_2_ signaling cascade (Kochar, et al., 2011 ▶; Murthy, 2006 ▶). Moreover, PKA acts as a downstream and upstream signaling cascade to induce relaxation via several mechanisms, in smooth muscle cells. 

As noted above, PKA and PKG act on upstream targets to regulate cAMP and cGMP levels by desensitizing receptors and stimulating PDE activities (Murthy, 2006 ▶). Moreover, a study demonstrated that the activation of ACh and His receptors is abolished by PKC inhibitors (Eto, et al., 2001 ▶).

In line with the aforementioned data, in our study, H89 potentiated smooth muscle relaxation induced by *M. recutita*, and this effect was decreased in the presence of ACh and L-arginine. 

Therefore, we suggest that specific interplay among different signaling cascades including PKA_2_, ACh, His, and adrenergic receptors, as well as the nitrergic pathway, is required to mediate the antispasmodic effect of *M. recutita* in rabbit intestinal smooth muscle. 

In this study, we were not able to determine the sequential pathway of NO and PKA_2 _activation in mediating the antispasmodic effects of *M. recutita* following the stimulation of ACh/His receptors. However, future studies should be conducted to provide sufficient evidence related to the downstream signaling pathways of *M. recutita* and focus on its possible link to ACh/His-receptors and the NO pathway.

In the present study, the concentration–response curves of *M. recutita* and its pure flavonoid fraction exhibited similar spasmolytic activity without significant changes in the EC_50_ values. Hence, the fundamental role of flavonoids in the anti-spasmodic effects of *M. recutita* was confirmed. Indeed, the total flavonoid fraction of *M. recutita *is used to prevent interfering actions of essential oils*. *Our previous study showed high amounts of apigenin and quercetin in the hydro-alcoholic extract of *M. recutita *(mazandarani et al., 2011 ▶). Thus, we suggest that the flavonoid fraction of *M. recutita* actively induces anti-spasmodic action. Moreover, a growing body of evidence suggests that apigenin and quercetin exhibit smooth muscle anti-spasmodic activities through a wide range of signaling cascades (Amira, et al., 2008 ▶; Gharzouli, et al., 2004 ▶). Consistent with our results, the myorelaxant activity of apigenin has been attributed to the inhibition of Ca^2+^ release or induction of eNOS and NO production in mouse gastric tissue (Chen, et al., 2010 ▶). In addition, Rotondo *et al*., showed significant NO production in response to an increase in intracellular calcium concentration by apigenin- and quercetin-induced gastric relaxation (Rotondo et al., 2009 ▶). Thus, we suggest that the total flavonoid fraction of *M. recutita* (apigenin, quercetin, etc.) mediates its inhibitory effect on smooth muscle contractions through blockade of calcium release and NO production. 

The myorelaxant activity *M. recutita* is observed as dual anti-spasmodic and spasmolytic effects. Considering the fundamental role of Ca^2+ ^channels as a possible end-effector step in the mechanism of action of *M. recutita*, we suggest that NO, His/ACh/ adrenergic receptors, and PKA_2_ are the main signaling components of *M. recutita* in the presence of spasmodic agents. Nevertheless, without analyzing the influence of *M. recutita* in the presence of several inhibitors or detecting direct kinase activity assay, we cannot determine whether the interactions among multiple mediators take place in parallel or in sequence. Meanwhile, further study is required to elucidate the mechanism of action of the various flavonoid components of *M. recutita* and the contributions of various signaling processes in the curative properties of this plant when uses in the treatment of gastrointestinal motility disorders. 
